# Bridging the Gap: The Role of Non-Invasive Respiratory Supports in Weaning from Invasive Mechanical Ventilation

**DOI:** 10.3390/jcm14207443

**Published:** 2025-10-21

**Authors:** Giulia Panzuti, Lara Pisani, Stefano Nava

**Affiliations:** 1 Alma Mater Studiorum, Department of Medical and Surgical Sciences (DIMEC), University of Bologna, 40126 Bologna, Italy; 2 Respiratory and Critical Care Unit, IRCCS Azienda Ospedaliero Universitaria di Bologna, 40138 Bologna, Italy

**Keywords:** acute respiratory failure, ARF, critical care, high-flow nasal cannula, HFNC, non-invasive respiratory supports, NIRS, non-invasive ventilation, NIV, weaning

## Abstract

Weaning from invasive mechanical ventilation (IMV) is a key element in the management of critically ill patients, encompassing the entire process of discontinuing IMV. Despite its importance, considerable uncertainties remain regarding the optimal strategies to achieve successful weaning. Early weaning is crucial, as IMV is associated with complications related to high mortality rates, such as prolonged weaning and intubation-associated pneumonia (IAP). This review aims to highlight the role of non-invasive respiratory supports (NIRSs), including non-invasive ventilation (NIV) and high-flow nasal cannulas (HFNCs), as a therapeutic bridge between IMV dependency and spontaneous breathing. NIV and HFNCs are recommended to prevent post-extubation respiratory failure (PERF) in high-risk and low-risk patients, respectively, and their combination appears effective in high-risk populations. On the other hand, NIV is not advised in established non-hypercapnic PERF, as it may increase mortality by delaying intubation; however, it can facilitate extubation in patients with hypercapnic respiratory failure. NIRSs may also benefit patients at high risk of post-operative pulmonary complications such as acute respiratory failure (ARF), with either NIV or HFNCs being appropriate. In light of this evidence, appropriate NIRSs selection and application may be pivotal in achieving successful weaning and better outcomes in critically ill patients.

## 1. Introduction

Weaning from invasive mechanical ventilation (IMV) is a key element in the management of critically ill patients with respiratory failure admitted to intensive care units (ICUs) worldwide. It refers to the full process of discontinuing IMV and, despite its clinical relevance, there remains considerable uncertainty about the optimal strategies to guide and optimize this transition. Initiating weaning as early as possible is essential, as prolonged exposure to IMV increases the risk of complications associated with higher mortality, such as intubation-associated pneumonia (IAP) and prolonged weaning failure [1,2,3]. Moreover, endotracheal intubation (ETI) can cause aspiration by impairing swallowing and airway defences, may damage the tracheal mucosa leading to bleeding or trauma, and often induces discomfort, which may require sedatives to manage anxiety and ensure compliance [4,5,6]. From a healthcare cost perspective, managing a patient on IMV is significantly more resource intensive [7,8].

Non-invasive respiratory supports (NIRSs), such as non-invasive ventilation (NIV) and high-flow nasal cannulas (HFNCs), play an important role in the weaning process, helping to bridge the gap between complete dependence on IMV and the reestablishment of spontaneous breathing.

This review aims to highlight the role of NIRSs in the weaning process and to guide clinicians toward best practices that promote improved patient outcomes. The focus will be limited to intubated patients; tracheostomized patients will not be specifically addressed.

A Medline search was performed to identify relevant literature on weaning from IMV and the role of NIRSs in this context, using combinations of MeSH terms and keywords, including ‘mechanical ventilation’, ‘weaning’, ‘non-invasive ventilation’, ‘high-flow nasal cannula’, and ‘post-extubation respiratory failure’.

## 2. Weaning Overview

### 2.1. Steps of the Weaning Process

**Figure 1:**
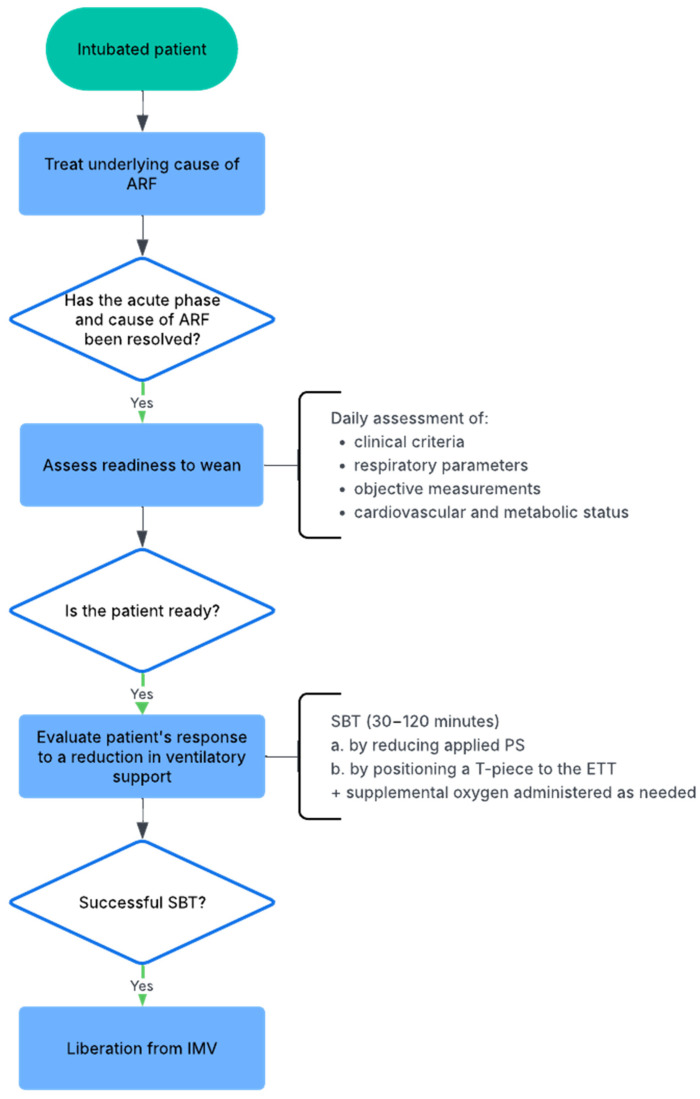
Flowchart of the weaning process from invasive mechanical ventilation (IMV). ARF: acute respiratory failure; SBT: spontaneous breathing trial; PS: pressure support; ETT: endotracheal tube; IMV: invasive mechanical ventilation.

Figure 1 illustrates the main steps in the weaning process, which should ideally be preceded by the management—though not necessarily the complete resolution—of the primary cause underlying the respiratory failure that required the patient’s intubation [9,10].

Despite evidence-based recommendations [9], weaning is often delayed, leading to increased patient discomfort, a higher risk of complications, and greater healthcare costs [11,12,13,14], especially in patients with prolonged weaning. To prevent delayed weaning, readiness should be assessed at least once daily. Ely et al. demonstrated that this strategy predicts successful extubation, reduces the risk of prolonged mechanical ventilation, and improves survival to discharge [15]. There are several criteria that can serve as indicators of readiness for a weaning trial, encompassing both clinical assessment and objective physiological measurements. Clinically, the patient should present with an adequate cough reflex, absence of excessive tracheobronchial secretions, and resolution of the primary cause of the acute illness that necessitated ETI. Objective measurements supporting readiness to wean include stable cardiovascular and metabolic status: peripheral oxygen saturation (SpO_2_) greater than 90% while receiving a fraction of inspired oxygen (FiO_2_) ≤ 0.4 and a positive end-expiratory pressure (PEEP) ≤ 8 cmH_2_O. Additional criteria include the absence of significant respiratory acidosis, a sufficient level of consciousness (e.g., arousable, Glasgow Coma Scale ≥ 13, and no continuous sedative infusions) to ensure airway protection, a tympanic temperature between 36 °C and 38 °C, no major electrolyte imbalances, and a haemoglobin concentration of at least 70–80 g/L [9,10,16,17]. In addition to these readiness criteria, predictors of weaning success comprise satisfactory pulmonary mechanics, including a respiratory rate (RR) ≤ 35 breaths per minute, maximal inspiratory pressure (MIP) ≤ −20 to −25 cmH_2_O, tidal volume (Vt) > 5 mL/kg of predicted body weight, vital capacity (VC) > 10 mL/kg, and a rapid shallow breathing index (RSBI)—calculated as RR divided by Vt—≤105 breaths per minute per litre [9,10,18,19,20]. Moreover, Perren et al. demonstrated that extubation success was more frequent in patients who were confident in their subjective perception of autonomous breathing—feeling capable of breathing without mechanical support—compared to those who were not confident [21]. Despite this evidence, patients’ ability to breathe independently is often underestimated in clinical practice, as evidenced by the fact that approximately half of unplanned self-extubations is successful and do not require reintubation [22].

When the patient meets these criteria, it is possible to try a spontaneous breathing trial (SBT) to evaluate the response to a reduction in ventilatory support [9]. The SBT can be performed by reducing the applied airway pressure using pressure support (PS) between 5–10 cmH_2_O, or by disconnecting the patient from the ventilator and attaching a T-piece to the endotracheal tube (ETT), with supplemental oxygen administered as needed to maintain adequate SpO_2_ and partial pressure of arterial oxygen (PaO_2_) levels. PS and T-piece SBTs do not appear to present significant differences in terms of weaning success, ICU mortality, ventilator-associated pneumonia (VAP), and ventilator-free days [23,24,25,26,27]. However, PS reduces respiratory effort compared to the T-piece but may underestimate the actual post-extubation workload [28]. In contrast, the T-piece and continuous positive airway pressure (CPAP) at 0 cmH_2_O appear to better reflect the patient’s physiological condition after extubation [29]. The usual duration of SBTs is at least 30 min and may be extended up to 120 min in patients considered at high risk of extubation failure [18,30]. More recently, a randomized controlled trial (RCT) demonstrated that a 30 min PS trial led to higher extubation success and lower hospital mortality compared to a 120 min T-piece trial in patients who had been mechanically ventilated for at least 24 h [31]. An SBT is considered failed if any form of clinical instability occurs during the trial (i.e., haemodynamic compromise, cardiac arrhythmias, tachypnoea, hypoxia or hypercapnia on blood gases, increased respiratory effort, altered mental status, agitation, or anxiety).

The optimal combination of screening frequency and SBT technique has been the subject of recent studies. Burns et al. found no overall difference in extubation time between screening frequency (once daily vs. more frequent) or SBT technique (PS vs. T-piece), although more frequent screening with pressure supported SBT unexpectedly delayed extubation [32]. The SPEED UP trial showed that, in low- or intermediate-risk patients with planned HFNCs, aggressive screening (less stringent criteria: PaO_2_/FiO_2_ > 180 on PEEP 10 cmH_2_O and FiO_2_ 50%) with preventive PEEP at 10 cmH_2_O until the SBT, combined with a conservative SBT (PS 5 + PEEP 0), shortened the time to extubation without increasing reintubation rate [33].

Finally, extubation should be performed in all patients who have successfully completed an SBT [34,35]. The latest ATS/ACCP guidelines conditionally recommend performing a cuff-leak test (CLT), involving deflation of the ETT cuff to assess airway patency, in patients at high risk of post-extubation stridor [36,37]. If the cuff-leak volume is low, systemic steroids should be administered at least 4 h before extubation to reduce the risk of upper airway obstruction [36,38,39,40].

Although simple, non-invasive, and inexpensive, the CLT has limited accuracy, with high specificity but only moderate sensitivity for post-extubation airway obstruction [41]. It can detect laryngeal oedema after prolonged ETI but does not reliably predict reintubation [42]. Recently, the COMIC pilot study found that sharing CLT results with the healthcare team did not affect post-extubation outcomes, including post-extubation stridor, reintubation, or emergency surgical airway interventions [43].

### 2.2. Weaning Success and Failure: Definitions and Patients’ Classification

The Statement of the Sixth International Consensus Conference on Intensive Care Medicine defined weaning success as extubation followed by the absence of ventilatory support for at least 48 h, and weaning failure as failure of the SBT, reintubation, resumption of ventilatory support, or death within 48 h [9]. Patients are classified into three groups based on weaning difficulty and duration: group 1 ‘simple weaning’ (success at first SBT, ~69% of patients), group 2 ‘difficult weaning’ (up to 3 SBTs or ≤7 days, ~20%), and group 3 ‘prolonged weaning’ (>3 SBTs or >7 days, ~15%) [9]. This classification has certain limitations related to the inability to categorize patients not undergoing an SBT because of their clinical condition or because of alternative weaning strategies and those who fail to wean.

Recently, Béduneau et al. proposed an evolution of the 2005 International Consensus Classification through the WIND (weaning according to a new definition) study. In this new system, patients are divided into four groups: ‘no weaning’ group (no separation attempt), group 1 ‘short weaning’ (≤1 day, ~60% of patients), group 2 ‘difficult weaning’ (2–7 days, ~20–30%), and group 3 ‘prolonged weaning’ (>7 days, ~10–15% of patients), with the latter further subdivided into successful (3a) and unsuccessful (3b) liberation from mechanical ventilation [44]. Successful weaning was redefined as extubation or ICU discharge without death or reintubation within 7 days, irrespective of post-extubation NIV [44]. These new classification and definitions, unlike the 2005 International Consensus Classification, allow the allocation of all patients into clinically meaningful groups and better reflects differences in morbidity and mortality, which increase after the first failed weaning attempt [44,45]. Table 1 provides a summary of the key differences between the two classifications.

## 3. Peri-Extubation Period

### 3.1. Extubation-Related Pathophysiological Changes

After extubation, patients experience multiple changes in airway dynamics and respiratory function due to the shift from positive-pressure ventilation to spontaneous, unassisted breathing. These changes can represent the underlying substrate for extubation failure, particularly in high-risk patients [46].

Among changes in airway status, upper airway obstruction is a significant cause of extubation failure (2–16% of ICU patients), most often resulting from laryngeal oedema due to mechanical trauma related to ETI [39,41]. Following ETT removal, airway narrowing increases airway resistance and may lead to respiratory distress [39,47,48], which provides the rationale for the guideline recommendation to perform a CLT [38]. Impairment in airway competence can also be related to the presence of abundant tracheobronchial secretions at the time of extubation in patients with a weak or ineffective cough [46].

Regarding lung aeration, the loss of positive intrathoracic pressure leads to alveolar derecruitment, increased work of breathing (WOB) due to reduced lung compliance, and hypoxemia in case of pre-existing atelectasis or underlying respiratory disease [49,50,51]. Pronounced negative swings in intrathoracic pressure related to the patient’s inspiratory effort can occur when discontinuing IMV, and this can contribute to cardiac overload—particularly concerning in patients with heart failure—by increasing left ventricular transmural pressure and afterload, as well as venous return and right ventricular preload [46]. Neuromuscular function can also be affected after extubation, as the respiratory muscle load increases following the removal of ventilatory support. In cases of prolonged IMV, patients may develop diaphragmatic dysfunction associated with excessive assistance, which during unassisted breathing after extubation can lead to an imbalance between the respiratory muscles capacity and the respiratory load, clinically manifesting as respiratory distress [52].

### 3.2. Post-Extubation Respiratory Failure (PERF)

The need for reintubation within hours or days after planned extubation is usually defined as extubation failure [53]. The timeframes used to define extubation failure vary, ranging from 48 h [18,54,55] to 72 h [56,57,58,59] and up to 7 days [60,61]. This occurs in 10–20% of patients who meet all weaning criteria and successfully complete an SBT [18,54,55,56,57,58,59,62,63]. Patients in whom extubation fails require longer periods of IMV and have mortality rates ranging from 20% to 50% [18,54,55,56,58,63,64]. Moreover, evidence suggests that extubation failure is associated with reduced survival, regardless of the severity of the underlying illness [58,63].

### 3.3. Risk Factors for Extubation Failure

There are many factors considered to be associated with an increased risk of extubation failure [65].These can be divided into three main categories: patient-related characteristics (e.g., advanced age, comorbid cardiac or respiratory disease, impaired cough) [58,66,67,68]; factors related to the underlying cause of the acute illness that required ETI (e.g., neurological disease, compromised airway patency and ability to manage endobronchial secretions, severity of illness, prolonged or difficult weaning, cardiogenic respiratory failure, pneumonia) [56,58,59,67,68]; and functional respiratory parameters reflecting the patient’s ventilatory capacity and respiratory drive (e.g., RR, RSBI, MIP, peak expiratory flow, VC, P0.1, P0.1/MIP ratio) [9,18,69,70,71].

## 4. State of the Art: Available NIRSs Interventions After Extubation

Given all the pathophysiological changes related to extubation and the high mortality rates associated with extubation failure, it is important to identify patients at risk of PERF to provide them with the most appropriate respiratory support strategy and improve their outcomes. In addition to conventional oxygen therapy (COT), post-extubation support can be provided through two main strategies, HFNCs and NIV, each with specific indications and potential benefits depending on the patient’s risk profile and clinical condition.

After a planned extubation, COT is administered when supplemental oxygen is required based on PaO_2_ or SpO_2_ values, using nasal prongs or face masks. However, this strategy allows for the delivery of only limited oxygen flows—up to a maximum of 15 L/min—which may be insufficient for patients experiencing PERF. Additionally, these patients often suffer from very high RRs, and as a result, ambient air dilutes the supplied oxygen, significantly reducing the effective FiO_2_ [72].

The role of NIRSs has been investigated in the prevention and treatment of PERF, the facilitation of extubation, and the management of acute respiratory failure (ARF) in the postoperative setting. Evidence-based guidelines relevant to these clinical contexts are summarized in Table 2.

### 4.1. NIV

NIV is a form of mechanical ventilation delivered through an interface that does not pass beyond the glottis, aimed at partially replacing the function of the inspiratory muscles and improving alveolar ventilation (Valv) and oxygenation. It is frequently administered using a bilevel pressure-targeted ventilation mode, which allows the delivery of a PS above a set PEEP during each patient-triggered breath. PEEP helps alveolar recruitment, increases aerated lung volume, and reduces ventilation/perfusion mismatch, thereby improving hypoxemia by preventing alveolar collapse and airway closure [77]. PS leads to an increase in Vt and Valv—resulting in improved gas exchange—and to a reduction in WOB, which is further decreased by PEEP in patients with dynamic hyperinflation (DH) and auto-PEEP by lowering the inspiratory threshold load and elastic load through increased respiratory system compliance [78,79,80]. Bilevel pressure-targeted ventilation mode can compensate for air leaks by adjusting the delivered inspiratory flow. Oronasal and full-face masks are the most commonly used interfaces. An important factor for a successful intervention is optimizing patient–ventilator interaction by adjusting settings such as cycling criteria, as suboptimal configurations may lead to asynchronies that can compromise the effectiveness and outcomes of NIV [81].

Notably, the use of NIV carries fewer risks than IMV. In fact, the risks associated with IMV are primarily due to the presence of an artificial airway, including a higher incidence of nosocomial pneumonia (NP), increased risk of aspiration, tracheal injury, greater patient discomfort, and frequent need for sedatives [4,5,6,82].

NIV, however, presents some issues related to its application, as patient’s discomfort can lead to non-compliance and, consequently, to interface displacement with air leaks and patient-ventilator asynchronies that limit the benefits of applied pressures and may result in a reduced number of hours of therapy [83]. To improve patient tolerance, clinicians should consider selecting the most suitable interface and applying active humidification [84]. In addition, NIV can be psychologically challenging for patients, often causing anxiety or feelings of claustrophobia. Panic can be reduced by clearly explaining the procedure, having a family member or staff member present, or using interfaces that cover less of the face [85,86]. In general, helmets may help reduce claustrophobia, prevent skin ulcers, enhance comfort, and facilitate the use of high PEEP with fewer issues related to air leaks [84]; however, these effects have not been specifically studied in the post-extubation setting. Moreover, positive pressure can cause gastric insufflation and abdominal distension, increasing the risk of vomiting and aspiration during NIV. This risk can be reduced by limiting sedatives, keeping the head of the bed elevated, and using a nasogastric tube when prolonged NIV is required [87].

### 4.2. HFNC

HFNCs deliver a heated and humidified mixture of air and oxygen at high flow rates, with a FiO_2_ ranging from 0.21 to 1.0.

HFNCs offer several physiological benefits: they provide a stable FiO_2_ by delivering a flow that matches or exceeds the patient’s peak inspiratory flow; the delivery of heated and humidified gas may improve mucociliary clearance and promote the fluidification and clearance of endobronchial secretions, as suggested by preclinical and circumstantial evidence [88,89,90]; they increase the fraction of minute ventilation by washing out anatomical dead space and carbon dioxide (CO_2_), leading to a reduced RR; and they decrease the patient’s inspiratory effort [91,92]. Vieira et al. demonstrated that, in a bench model, end-expiratory nasopharyngeal pressure increased to approximately 4 cmH_2_O at a flow rate of 60 L/min. In healthy volunteers receiving HFNCs at the same flow with their mouths closed, nasopharyngeal pressure reached a median value of 6.8 cmH_2_O, indicating that the positive pressure effects are influenced by both flow rate and mouth position. Nevertheless, despite these experimental findings showing elevated nasopharyngeal pressures, the existence of a clinically significant PEEP effect associated with HFNCs remains highly uncertain [93]. In addition, HFNCs improve patient comfort through the use of nasal prongs, which—unlike face masks—are often better tolerated by claustrophobic patients, cause fewer skin lesions and allow the patient to freely perform normal daily activities such as eating and talking [94]. These factors may contribute to improved adherence to therapy.

## 5. Role of NIRSs in Facilitating Extubation

The latest ERS/ATS guidelines provide a conditional recommendation for the use of NIV to facilitate weaning from IMV in patients with hypercapnic ARF, as its application in this population is associated with lower mortality rates (risk ratio 0.54, 95% CI 0.41–0.70), reduced rates of weaning failure (risk ratio 0.61, 95% CI 0.48–0.79), and a decreased incidence of VAP (risk ratio 0.22, 95% CI 0.15–0.32) [73]. This recommendation is supported by the results of 16 RCTs identified in a Cochrane review [95]. A recent systematic review with meta-analysis including 11 studies showed that prophylactic NIV reduced reintubation rates, ICU and hospital length of stay, and mortality, further supporting its use in clinical practice [96]. The physiological basis of NIV use in hypercapnic patients, often affected by chronic obstructive pulmonary disease (COPD), lies in their impaired pulmonary mechanics, characterized by increased auto-PEEP, elevated airway resistance, reduced pressure-generating capacity of the respiratory muscles, and DH [97,98]. In such clinical scenarios, NIV improves breathing mechanics and gas exchange by providing inspiratory support, which reduces diaphragmatic WOB, relieves respiratory muscle fatigue, and lowers the elastic load caused by DH [78,79]. In this regard, Vitacca and colleagues demonstrated that non-invasive pressure support ventilation (PSV) provides clinical and physiological responses similar to those of IMV when the same inspiratory and expiratory pressures are applied, highlighting comparable diaphragmatic energy expenditure per breath and per minute in both modalities [99].

The first RCT in this field was conducted by Nava et al. and included 50 COPD patients who required ETI for hypercapnic ARF secondary to an acute exacerbation of COPD, randomized to either extubation followed by continuous NIV or continuation of IMV after 48 h of IMV and failure of an SBT. The first group showed a shorter ICU stay, a lower incidence of NP, and improved 60-day survival rates compared with IMV-sustained weaning [100].

Evidence on the efficacy of NIV in hypoxemic patients is limited. One study that randomized 20 hypoxemic intubated patients to either NIV or conventional weaning did not find significant differences in arterial blood gas values or weaning success. However, the number of IMV days was significantly lower in the NIV group [101].

In summary, NIV is an effective strategy to facilitate extubation and weaning in hypercapnic patients—particularly those with COPD—by improving respiratory mechanics and reducing IMV duration, ICU stay, and mortality, whereas evidence for its benefit in hypoxemic patients remains limited and warrants further investigation.

## 6. NIRSs as a Strategy to Prevent PERF

NIRSs can be employed to prevent PERF in patients with a planned extubation. For this purpose, it is necessary to identify risk categories for extubation failure in order to ensure that patients receive optimal respiratory support and care.

Traditionally, a patient can be classified as high-risk for extubation failure if they have at least one high-risk factor included in any of the available models. Simplifying the risk factors for extubation failure illustrated in paragraph 3.3, evidence suggests that patients over 65 years of age with underlying cardiac or respiratory disease can be considered high-risk, presenting a re-intubation rate of over 30% if both comorbidities are present, and over 20% if only one is [102]. Alternatively, there is an 11-factor model that takes into account age, comorbidities integrated in the Charlson index including at least moderate COPD, acute illness characteristics such as prolonged IMV and acute heart failure, and the clinical condition at the time of extubation [66,75]. A limitation of this evaluation is that it requires a significant amount of time from clinicians to be calculated and may overestimate the risk by considering all the included factors. A large post hoc study including patients with varying risk showed that both the 3- and 11-factor models have low diagnostic accuracy, with the 3-factor model showing lower sensitivity (62.96% vs. 84.20%) and only a slight advantage in specificity (44.75% vs. 31.46%) [103].

In clinical practice, existing risk-stratification tools may help identify patients at high risk of extubation failure but should be applied cautiously given their limited diagnostic accuracy. Future research should focus on developing and validating more reliable, practical, and time-efficient models—potentially leveraging artificial intelligence—to integrate patient comorbidities and acute illness characteristics.

### 6.1. NIRSs for Preventing PERF in High-Risk Patients

There are two main RCTs supporting the use of NIV after planned extubation in high-risk patients to prevent PERF. Firstly, Nava and colleagues conducted an RCT involving 97 patients considered at risk for developing PERF and randomized them to receive either NIV for ≥8 h per day during the first 48 h, or standard medical therapy, with the primary endpoint being reintubation within 72 h and PERF. They found that the NIV group had a lower reintubation rate and a reduced risk of death in the ICU [68]. The second study, which enrolled 106 high-risk mechanically ventilated patients and randomized them to receive either NIV or oxygen therapy alone, showed that NIV was independently associated with a lower risk of PERF and led to a reduction in 90-day mortality [104]. Given this evidence, the ERS/ATS guidelines provide a conditional recommendation for the use of NIV to prevent PERF in high-risk patients, as it has been shown to reduce mortality rates (risk ratio 0.41, 95% CI 0.21–0.82; moderate certainty) and the need for ETI (risk ratio 0.75, 95% CI 0.49–1.15) [73].

A large noninferiority RCT comparing conventional NIV and HFNCs initiated 24 h after extubation in high-risk patients demonstrated that HFNCs met noninferiority criteria, with reintubation rates at 72 h of 19% and 23%, respectively [75]. However, a French multicenter RCT involving 641 patients at high risk of extubation failure randomized to an HFNC alone or an HFNC alternating with NIV after extubation (HIGH-WEAN trial) demonstrated that the HFNC + NIV group had a significantly lower risk of reintubation compared with an HFNC alone [76].

The clinical relevance of this finding becomes particularly pronounced when obese patients are taken into consideration. It is known that obesity causes alterations in static lung volumes, with an exponential reduction in expiratory reserve volume (ERV) as obesity severity increases, while residual volume (RV) is typically preserved, resulting in a decreased functional residual capacity (FRC) [105]. It has been further observed that the obesity-related reduction in ERV leads to a decrease in PaO_2_, with a linear relationship to increasing body mass index (BMI), independently of hypoventilation [106]. A recent systematic review and network meta-analysis evaluating NIRS therapies after extubation in critically ill obese adults highlighted that NIV alone or combined with an HFNC reduces reintubation rates compared to COT and an HFNC alone and, furthermore, that NIV alone or NIV + HFNC improves survival by reducing mortality rates compared to an HFNC [107].

Overall, NIV—alone or combined with an HFNC—effectively prevents PERF and reduces reintubation and mortality rates in high-risk patients, including obese individuals, although careful attention to patient tolerance and interface management is required. Future research should specifically assess whether HFNCs can reduce the WOB compared with COT during breaks from NIV, as suggested by the results of a study that systematically applied HFNCs between NIV sessions [108]. However, since the study was designed with a different primary aim, these results should be interpreted cautiously.

### 6.2. NIRSs for Preventing PERF in Unselected, Non-High-Risk Patients

The use of NIV in unselected, non–high-risk patients is not recommended [73]. Supporting this, a 1999 study involving 93 patients who underwent either planned or unplanned extubation and were randomized to receive preventive NIV or standard treatment showed no significant difference in the reintubation rate between the two groups [109]. Similarly, Su et al. studied 406 unselected patients extubated after passing a SBT and, as in the previously cited RCT, found no difference in either the reintubation rate or ICU mortality between the NIV and standard treatment groups [110].

In this context, the use of an HFNC as a form of NIRS plays an important role. According to the physiological benefits highlighted in Section 4.2, HFNCs may help prevent hypoxemic episodes after extubation, decrease RR, facilitate secretion clearance, and reduce atelectasis compared to COT. Hernández et al., analysing data from 527 adult critically ill patients at low risk of reintubation who fulfilled criteria for planned extubation and were randomized to receive either an HFNC or COT for 24 h post-extubation, showed that HFNCs significantly reduced the risk of reintubation within 72 h compared to COT in this low-risk population [66]. Additionally, another RCT comparing HFNCs with COT showed that HFNCs provided better oxygenation at the same FiO_2_ after extubation and were associated with improved comfort, fewer desaturations, and fewer interface displacements [111]. The ERS clinical practice guidelines provide a conditional recommendation for the use of HFNCs over COT in nonsurgical patients at low to moderate risk of extubation failure [74]. This is based on findings showing a reduction in reintubation rates (risk ratio 0.62, 95% CI 0.38–1.01; risk difference −5.1%, 95% CI −8.2% to 0.1%; moderate certainty) and a decreased need for escalation to NIV (risk ratio 0.38, 95% CI 0.17–0.85; risk difference −9.4%, 95% CI −12.5% to −2.3%; moderate certainty) [74]. However, Maggiore et al. conducted a multicenter RCT in 494 post-extubation patients with a PaO_2_/FiO_2_ ratio ≤ 300 mmHg and found that reintubation rates were similar between HFNCs and COT, although HFNCs reduced the need for rescue NIV [112].

These findings indicate that NIV is not beneficial in unselected, low-risk patients, whereas HFNCs reduce reintubation and the need for escalation to NIV, supporting their use in nonsurgical patients at low to moderate risk of extubation failure. Nevertheless, evidence on HFNC use after extubation in low-risk patients remains conflicting, likely due to the blurred distinction between preventive and therapeutic strategies, further complicated by the frequent occurrence of residual hypoxemia at extubation. Future research should explore patient-tailored HFNC strategies by clearly distinguishing preventive from therapeutic applications to ensure optimal NIRS management.

## 7. Therapeutic Use of NIRSs in PERF

According to ERS/ATS guidelines recommendations, NIV should not be used for treating PERF in non-hypercapnic patients as its application seems to be associated with higher mortality rates (risk ratio 1.33, 95% CI 0.83–2.13, low certainty) [73]. Two RCTs on patients experiencing, respectively, respiratory distress and ARF within 48 h after extubation, who were randomized to receive either NIV or standard treatment with reintubation if needed, did not find any beneficial effect of NIV on reintubation rates, ICU or hospital mortality, or length of ICU and hospital stay [113,114]. On the contrary, higher mortality rates were observed in the NIV group, possibly due to delayed ETI [114].

No studies have been reported so far investigating the role of HFNCs in patients experiencing PERF.

In summary, current evidence indicates that NIV is not recommended for PERF in non-hypercapnic patients due to lack of efficacy and possible increased mortality, while the role of HFNCs in this setting remains unexplored.

The optimal NIRS strategy for patients with established PERF remains unknown, and future efforts to address this gap include a large-scale ongoing RCT (NCT05686850) investigating whether alternating NIV with HFNCs, compared with HFNCs alone, can reduce mortality in ICU patients with PERF.

## 8. NIRSs in the Post-Operative Setting

Patients may experience a range of post-operative pulmonary complications, from atelectasis to acute respiratory distress syndrome (ARDS). ARF typically occurs within 7 days after surgery, and its development is influenced by several factors, including the type and duration of the surgical procedure, the use of anaesthesia and IMV, as well as patient-related factors such as age, comorbidities, and lifestyle. These complications are associated with increased morbidity, mortality, and length of hospital stay [115,116,117].

Unlike what has been previously stated for non-surgical patients, NIV is conditionally recommended for use in post-operative patients experiencing ARF, as it has been shown to reduce mortality rates (risk ratio 0.28, 95% CI 0.09–0.84; moderate certainty), the need for intubation (risk ratio 0.27, 95% CI 0.12–0.61; low certainty), and the incidence of NP (risk ratio 0.20, 95% CI 0.04–0.88; very low certainty) [73]. There is evidence of NIV-related improvements compared to COT in clinical outcomes in patients who have undergone thoracic surgery, abdominal surgery, or solid organ transplantation [118,119,120,121,122]. Results of the NIVAS multicenter RCT, in which 293 patients who developed ARF after abdominal surgery were randomized to receive either NIV or COT, show that the NIV group experienced a significantly lower reintubation rate [121]. A systematic review reported that postoperative NIV improves lung volumes and gas exchange and may reduce pulmonary complications, reintubation rates, and hospital length of stay [123]. Supporting this, a recent network meta-analysis found that only NIV significantly reduced reintubation rates and mortality compared with COT, without causing significant patient discomfort [124]. Interestingly, a large RCT (the Bilevel Positive Airway Pressure Versus Optiflow non-inferiority trial) comparing outcomes in 830 patients with or at risk of hypoxemic ARF after cardiothoracic surgery, randomized to receive either NIV or HFNCs, demonstrated that the effectiveness in avoiding reintubation was comparable between HFNCs and NIV when used as therapeutic strategies [125]. Therefore, the latest ERS guidelines suggest using either HFNCs or NIV in post-operative patients at high risk of respiratory complications, although the point estimate for mortality favours NIV over HFNCs—despite considerable uncertainties [74]. This suggests that HFNCs may represent a first-line NIRS strategy for this patient subpopulation.

Finally, when considering the prophylactic role of NIRSs in preventing extubation failure, a recent network meta-analysis [126] showed that in post-surgical ICU patients, only an HFNC significantly reduced the incidence of re-intubation compared to COT (OR 0.13; 95% CI: 0.04–0.45; *p* = 0.001).

Overall, the evidence indicates that in post-operative patients, both NIV and HFNCs are effective for treating ARF, with NIV potentially achieving a greater reduction in reintubation rate. Current limitations relate to most studies assessing all-cause reintubation during hospital stay without distinguishing between different causes of post-operative ARF, and to recent meta-analyses comprising studies published over a 25-year period. Future research should therefore target more carefully selected patient populations. Moreover, extending the use of machine learning models—such as the recently validated CatBoost model [127]—to post-operative patients undergoing general anesthesia may facilitate the timely identification of patients at high risk of NIV failure.

## 9. Conclusions

In conclusion, NIRSs—such as NIV and HFNCs—are valuable strategies to be employed in the weaning process from IMV, an integral part of the management of critically ill patients with relevant impacts on prognosis and mortality. As outlined in this review, NIRSs can be useful in managing PERF in both acute and post-operative settings and in facilitating extubation. It is important to choose the right strategy for the right patient, based on current evidence and a multidimensional evaluation of the risk of extubation failure, considering both the clinical scenario and the patient’s characteristics, including comorbidities, the acute underlying pathology, and vital parameters. Although some risk categories are broadly defined, they do not always strongly predict extubation failure and should therefore be interpreted and used with caution to support clinical decision-making and help optimize patient care.

## Figures and Tables

**Table 1:** Comparison of weaning classifications: 2005 International Consensus [9] versus WIND Study [44].

Feature	2005 ICC [9]	WIND Study [44]
Definition of weaning success	Extubation followed by the absence of ventilatory support for ≥48 h	Extubation without death/reintubation within 7 days, or ICU discharge without IMV within 7 days (whichever comes first)
Definition of weaning failure	SBT failure, reintubation, resumption of ventilatory support, or death within 48 h	SBT failure, reintubation, resumption of ventilatory support, or death within 7 days
Classification basis	- SBT number - Days to successful weaning	Time elapsed from first separation attempt
Main groups	(1) Simple weaning: 1 successful SBT (2) Difficult weaning: ≤3 SBTs or ≤7 days from first SBT (3) Prolonged weaning: >3 SBTs or >7 days from first SBT	(0) No weaning (1) Short weaning: success or death ≤1 day from first separation attempt (2) Difficult weaning: weaning lasts 2–7 days (3) Prolonged weaning: >7 days after first separation attempt (3a) success (3b) still ventilated
Potential limitations	- Unclassified patients without SBT - Uncovered clinical scenarios (alternative weaning strategies, non-SBT weaning failure, tracheostomized patients not clearly included)	- Time-based categorization - Limited consideration of post-extubation interventions (e.g., COT, HFNCs, NIV)

ICC: international consensus classification; ICU: intensive care unit; IMV: invasive mechanical ventilation; SBT: spontaneous breathing trial; MV: mechanical ventilation; COT: conventional oxygen therapy; HFNC: high-flow nasal cannula; NIV: non-invasive ventilation.

**Table 2:** Guidelines [73,74] recommendations for non-invasive respiratory supports (NIRSs) use during weaning, post-extubation, and postoperative periods.

Indication	Risk Class	NIV	HFNC
To facilitate extubation	—	✓ if hypercapnic	✗
To prevent PERF	high-risk	✓	✗ *
	non-high-risk	✗	✓
To treat PERF	—	✗	—
To prevent/treat ARF in post-operative setting	high-risk of PPCs	✓	✓
	low-risk of PPCs	✗	✓

NIV: non-invasive ventilation; HFNC: high-flow nasal cannula; ✓: indicated; ✗: not indicated; —: evidence not available; PERF: post-extubation respiratory failure; ARF: acute respiratory failure; PPCs: post-operative pulmonary complications. * A large noninferiority RCT showed that an HFNC was noninferior to conventional NIV when initiated 24 h after extubation in high-risk patients, with similar reintubation rates (19% vs. 23%, respectively) [75]. However, the HIGH-WEAN trial demonstrated that combining an HFNC with NIV significantly reduced the risk of reintubation compared to an HFNC alone in high-risk patients [76].

## Data Availability

No new data were created or analyzed in this study. Data sharing is not applicable to this article.

## Abbreviations

The following abbreviations are used in this manuscript:

ARDS	Acute Respiratory Distress Syndrome
ARF	Acute Respiratory Failure
BMI	Body Mass Index
CLT	Cuff-Leak Test
CO_2_	Carbon dioxide
COPD	Chronic Obstructive Pulmonary Disease
COT	Conventional Oxygen Therapy
CPAP	Continuous Positive Airway Pressure
DH	Dynamic Hyperinflation
ERV	Expiratory Reserve Volume
ETI	Endotracheal Intubation
ETT	Endotracheal Tube
FiO_2_	Fraction of inspired oxygen
FRC	Functional Residual Capacity
HFNC	High-Flow Nasal Cannula
IAP	Intubation-Associated Pneumonia
ICU	Intensive Care Unit
IMV	Invasive Mechanical Ventilation
MIP	Maximal Inspiratory Pressure
NIRS	Non-Invasive Respiratory Support
NIV	Non-Invasive Ventilation
NP	Nosocomial Pneumonia
PaO_2_	Partial pressure of arterial oxygen
PEEP	Positive End-Expiratory Pressure
PERF	Post-Extubation Respiratory Failure
PS	Pressure Support
PSV	Pressure Support Ventilation
RCT	Randomized Controlled Trial
RR	Respiratory Rate
RSBI	Rapid Shallow Breathing Index
RV	Residual Volume
SBT	Spontaneous Breathing Trial
SpO_2_	Peripheral oxygen saturation
Valv	Alveolar Ventilation
VAP	Ventilator-Associated Pneumonia
VC	Vital Capacity
Vt	Tidal Volume
WOB	Work Of Breathing
